# Gingival mesenchymal stem cell‐derived exosomes are immunosuppressive in preventing collagen‐induced arthritis

**DOI:** 10.1111/jcmm.17086

**Published:** 2021-12-24

**Authors:** Xiaohong Tian, Wumei Wei, Yue Cao, Tianrang Ao, Feng Huang, Rabia Javed, Xiaohong Wang, Jun Fan, Yanhui Zhang, Yanying Liu, Laijun Lai, Qiang Ao

**Affiliations:** ^1^ Department of Tissue Engineering School of Intelligent Medicine China Medical University Shenyang China; ^2^ Cancer Hospital Chinese Academy of Medical Sciences & Peking Union Medical College Beijing China; ^3^ Department of Clinical Immunology Sun Yat‐sen University Third Affiliated Hospital Guangzhou PR China; ^4^ Department of Rheumatology and Immunology Peking University People's Hospital Beijing China; ^5^ Department of Allied Health Sciences University of Connecticut Storrs Connecticut USA; ^6^ National Engineering Research Center for Biomaterials Institute of Regulatory Science for Medical Device Sichuan University Chengdu China

**Keywords:** gingival mesenchymal stem cells, GMSC‐derived exosomes, NF‐κB, rheumatoid arthritis, Th17/Treg balance

## Abstract

Due to the unsatisfied effects of clinical drugs used in rheumatoid arthritis (RA), investigators shifted their focus on the biotherapy. Although human gingival mesenchymal stem cells (GMSC) have the potential to be used in treating RA, GMSC‐based therapy has some inevitable side effects such as immunogenicity and tumorigenicity. As one of the most important paracrine mediators, GMSC‐derived exosomes (GMSC‐Exo) exhibit therapeutic effects via immunomodulation in a variety of disease models, bypassing potential shortcomings of the direct use of MSCs. Furthermore, exosomes are not sensitive to freezing and thawing, and can be readily available for use. GMSC‐Exo has been reported to promote tissue regeneration and wound healing, but have not been reported to be effective against autoimmune diseases. We herein compare the immunomodulatory functions of GMSC‐Exo and GMSC in collagen‐induced arthritis (CIA) model and in vitro CD4^+^ T‐cell co‐culture model. The results show that GMSC‐Exo has the same or stronger effects compared with GMSC in inhibiting IL‐17A and promoting IL‐10, reducing incidences and bone erosion of arthritis, via inhibiting IL‐17RA‐Act1‐TRAF6‐NF‐κB signal pathway. Our results suggest that GMSC‐Exo has many advantages in treating CIA, and may offer a promising new cell‐free therapy strategy for RA and other autoimmune diseases.

## Graphical abstract

Gingival mesenchymal stem cell‐derived exosomes (GMSC‐Exo) ameliorate inflammation and bone erosion of metatarsophalangeal joint through regulating CD4^+^T cells, which mainly by suppressing the differentiation of Th1 and Th17 cells while promoting Treg cells differentiation. Simultaneously, GMSC‐Exo also inhibit IL‐17RA‐NF‐κB signal pathway to exert their therapeutic effects in collagen‐induced arthritis.

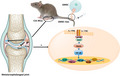

## 1 INTRODUCTION

Rheumatoid arthritis (RA) is a severe inflammatory autoimmune disease (AID) characterized by the infiltration of activated immune cells and the production of inflammatory factors that lead to chronic synovitis and progressive destruction of cartilage and bone in multiple joints.^1^ Though the pathogenesis of RA is not fully understood, studies show that it involves the abnormal activation of various immune cells, such as macrophagocyte, T and B cells, dendritic cells, and NK cells. Among these cells, CD4^+^ T cells play a key role in the development of inflammation, especially the helper T cell (Th cells) subsets Th1, Th17 cells and regulatory T cells (Treg).^1^ Furthermore, it has been previously reported that the imbalance in Th17/Treg is a decisive factor.^2^, ^3^, ^4^ Th17 cells secrete various pro‐inflammatory cytokines, mainly IL‐17, which could activate related signal transduction pathways (such as NF‐κB) and induce the production of pro‐inflammatory cytokines (such as tumour necrosis factor [TNF]‐α, IL‐6 and IL‐1β), chemokines and matrix metalloproteinases, leading to tissue invasion and destruction as well as damage of articular cartilage and bone.^5^ Currently, RA patients are normally treated with disease‐modifying antirheumatic drugs (DMARDs), including synthetic (or chemical) DMARDs (sDMARDS) and biological DMARDs (bDMARDs). The former is divided into conventional synthetic and targeted synthetic DMARDs (csDMARDs and tsDMARDs), while the latter is divided into biological original and biosimilar DMARDs (boDMARDs and bsDMARDs).^6^ But the traditional drug treatment can only relieve the symptoms, none is curative. In addition, some adverse reactions occur when these drugs are used for a long time.^1^ Therefore, it is urgent to seek novel approaches to improve present status for RA treatment.

Cell‐based therapeutic approaches are currently developed in RA, mainly mesenchymal stem cells (MSCs)‐based approaches. Human gingival mesenchymal stem cells (GMSC) exist in human gingiva and have been attracting increased attention due to their easier isolation, faster proliferation, stable phenotype and immunomodulatory capacities.^7^, ^8^, ^9^ In addition, GMSC have been reported to have preventive or therapeutic effects in several AIDs animal models and humanized animal models.^10^, ^11^, ^12^, ^13^, ^14^, ^15^, ^16^, ^17^

However, a particular problem in MSCs field is that after systemic transplantation, MSCs are rapidly trapped in the pulmonary vascular bed because of their large size.^18^, ^19^ Usually less than 1% of MSCs reach and are implanted in the target sites. Despite this, the therapeutic effect still be observed and was mainly attributed to paracrine actions and MSCs apoptosis.^20^, ^21^, ^22^ Indeed, apoptotic cells have been shown to treat ongoing experimental arthritis.^23^ As one of the most important paracrine mediators, MSC‐derived exosomes exhibit therapeutic effects via immunomodulation in a variety of disease models, bypassing potential shortcomings of the direct use of MSCs.^24^, ^25^, ^26^, ^27^, ^28^ Furthermore, exosomes are resistant to freezing and thawing, and can be readily available for use. GMSC‐derived exosomes (GMSC‐Exo) have been reported to promote tissue regeneration and wound healing,^24^, ^29^, ^30^ but have not been reported to be effective against autoimmune diseases. Importantly, it is reported that exosomes produced by MSCs pre‐stimulated by inflammatory factors have stronger immune regulation capabilities.^31^, ^32^, ^33^ Coincidentally, the microenvironment where GMSC are located is rich in food residues, microbial flora and saliva, which provides a natural inflammatory microenvironment for GMSC. This is the unique advantage of GMSC, so the GMSC‐Exo naturally have stronger immunoregulatory capabilities than other MSCs and could be the best choice for the treatment of RA.

In the present study, we demonstrate that administration of GMSC‐Exo/GMSC significantly alleviate the inflammation and bone erosion in collagen‐induced arthritis (CIA) model, and GMSC‐Exo have the same or stronger effect compared with GMSC. We further observed that GMSC‐Exo play a role by regulating the imbalance of Th17/Treg in vitro or in vivo, and inhibiting IL‐17RA‐Act1‐TRAF6‐NF‐κB signal pathway in vivo. In conclusion, our results suggest that GMSC‐Exo can be applied as a promising therapeutic approach for patients with RA and other autoimmune diseases.

## 2 MATERIALS AND METHODS

### 2.1 Mice

DBA/1J mice (male, 6–8 week old) were obtained from Nanjing Biomedical Research Institute of Nanjing University (Nanjing, China). C57BL/6J mice (male, 6–8 week old) were purchased from Liaoning Changsheng biotechnology company (Shenyang, China). All experiments using mice were performed in accordance with protocols approved by the Institutional Animal Care and Use Committee (IACUC) at China Medical University.

### 2.2 Isolation, culturing and characterization of human GMSC

Human tissue samples were obtained from discarded tissues of patients who had relatively healthy periodontium undergoing routine dental procedures and who provided informed consent in the Dental Division of the Third Affiliated Hospital at Sun Yat‐sen University. This study was carried out in accordance with the recommendations of the ethical review committee of clinical research of the Third Affiliated Hospital of Sun Yat‐sen University. Human GMSC were obtained by following the protocol described previously.^10^, ^11^ The cells were cultured with complete growth medium (MEM alpha [Gibco] supplemented with 10% foetal bovine serum [Gibco], 100 μg/ml penicillin and 100 μg/ml streptomycin [Gibco], 100 µM MEM non‐essential amino acids [Gibco], 550 µM 2‐ME [Sigma‐Aldrich], 10 mM HEPES, 1 mM sodium pyruvate, 2 mM l‐glutamine [Gibco]) at 37°C in a humidified tissue culture incubator with 5% CO_2_.

We characterized GMSC by detecting the stem cell phenotypic markers and multipotent differentiation properties. Sub‐cloning cultures were used to purify GMSC, and cells from the third passages were used in experiments. For GMSC characterization markers detection, GMSC were stained with mAbs for human CD34, CD44, CD45, CD73, CD90 and CD105 (Biolegeng) and assessed by flow cytometry.

For GMSC multipotent differentiation properties detection, osteogenic differentiation and adipogenic differentiation were used. For osteogenic differentiation, the GMSC were seeded in 6‐well plates (1 × 10^5^ cells/well; Corning) and incubated with 2 ml of complete growth medium (α‐DMEM). After GMSC reached 80%–90% confluency, the cell medium was replaced with osteogenic induction medium: α‐DMEM (Gibco) containing 10% FBS, 0.1 μM dexamethasone (Sigma‐Aldrich), 10 mM β‐glycerol phosphate (Sigma‐Aldrich) and 50 μg/ml L‐ascorbic acid (Sigma‐Aldrich). The medium was refreshed every 3 days. Two weeks later, the cells were fixed and assayed by alizarin red staining kit (Sigma‐Aldrich).

For adipogenic differentiation, the GMSC were cultured in adipogenic differentiation medium: α‐DMEM containing 500 nM isobutylmethylxanthine (Sigma‐Aldrich), 60 μM indomethacin (Sigma‐Aldrich), 500 nM hydrocortisone (Sigma‐Aldrich), 10 μg/ml insulin (Sigma‐Aldrich) and 100 nM L‐ascorbic acid phosphate. After 14 days, the cultured cells were stained with Oil Red‐O (Sigma‐Aldrich).

### 2.3 Production and characterization of GMSC‐Exo

**FIGURE 1:**
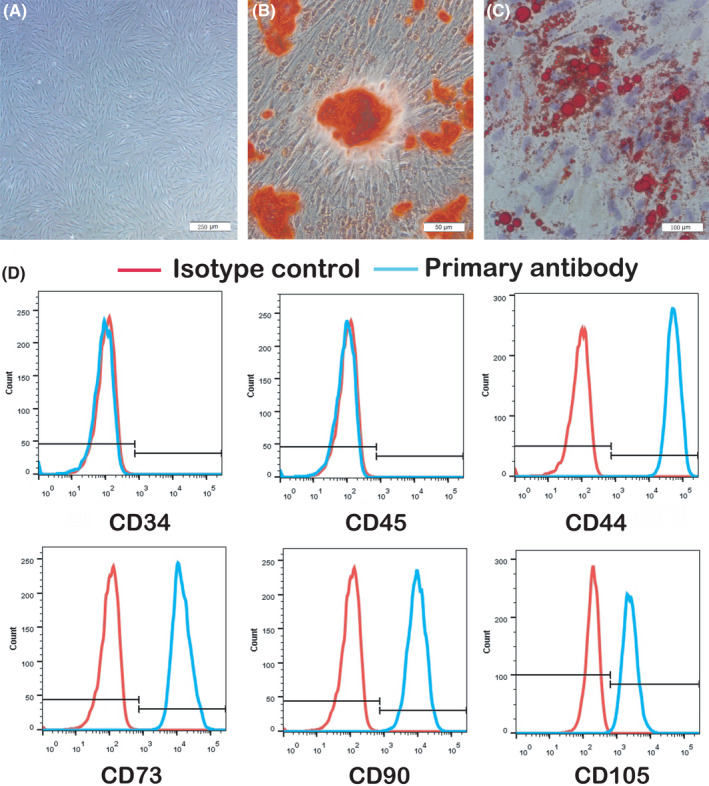
Characterization of GMSC and multipotent differentiation in vitro. (A) Representative image of GMSC at passage 3. Scale bar: 250 μm. (B) Osteogenic differentiation of GMSC. Alizarin red staining showed the mineralized nodule formed by GMSC. Scale bar: 50 μm. (C) Adipogenic differentiation of GMSC. Oil red staining showed the lipid droplet formed by GMSC. Scale bar: 100 μm. (D) Flow cytometric analysis showed that GMSC were positive for the surface molecules of CD44, CD73, CD90 and CD105 while negative for CD34 and CD45. Data are representative of three separate experiments. GMSC, gingival mesenchymal stem cells

Gingival mesenchymal stem cell were seeded at 5 × 10^5^ cells/ml on 10 cm dish with complete growth medium. When GMSC reached 80%–90% confluence, they were cultured in conditioned medium with exosomes‐free foetal bovine serum for 24 h, and then, the supernatants containing GMSC‐Exo were harvested. The GMSC‐Exo were extracted by differential ultracentrifugation. In brief, the supernatants were centrifuged at 300 g for 10 min and 2000 *g* for 10 min to remove the detached cells and cell debris/apoptotic bodies respectively. Then, the supernatant was centrifuged for 30 min at 10,000 g to remove microvesicles (MVs). After that, the clarified supernatant was centrifuged at 100,000 g for 70 min and the pellet on the bottom of the tube was GMSC‐Exo. Then, the pellet was washed with phosphate‐buffered saline (PBS) by centrifuging at 100,000 g for 70 min (Figure 1A).^34^ Finally, the concentrated GMSC‐Exo were suspended in PBS and determined using a BCA protein assay kit (Beyotime).

Size distribution of GMSC‐Exo was determined by nanoparticle tracking analysis (NTA) using a Zetasizer Nano ZS90 instrument as advised by the manufacturer (Malvern Zetasizer Software v7.11 PSS0012‐37 EN JP). Visualization of GMSC‐Exo was assessed by transmission electron microscopy (TEM, Hitachi HT7700, JP). GMSC‐Exo suspensions were loaded on Formvar‐coated grids and negatively stained with uranyl acetate for 15 min. Grids were observed using a microscope. In addition, CD9, CD63 and CD81 (ABclonal Technology) were measured by Western blot.

### 2.4 In vitro proliferation assay

To examine and compare the effects of GMSC‐Exo and GMSC on the proliferation of CD4^+^ T cells in vitro, we applied CFSE Cell Division Tracker Kit (Biolegend). Mouse CD4^+^ T cells were isolated from splenocytes by a CD4^+^ T Cell Isolation Kit (Miltenyi Biotec) according to the manufacturer's protocol and labelled with CFSE, then cultured alone or with GMSC‐Exo at the ratio of 10 µg/40 µg/90 µg: 10^6^ (GMSC‐Exo: CD4^+^ T cells), or with GMSC at the ratio of 1:1, 1:10 or 1:50 (GMSC: CD4^+^ T cells). The anti‐CD3 (5 µg/ml; Biolegend) was pre‐coated on the bottom of culture plate, and the anti‐CD28 (5 µg/ml; Biolegend) was added with GMSC‐Exo or GMSC. The GMSC‐Exo/GMSC were added for 3 days, then the cells were analysed using a BD FACSCelesta flow cytometer and the levels of Th1, Th17, and Treg cytokines (IFN‐γ, IL‐17A, and IL‐10) (Nos.430807, 432507, 431417, Mouse ELISA Kit; Biolegend) were detected by ELISA according to the manufacturer's protocols.^35^

### 2.5 Induction and treatment of CIA

For CIA induction, bovine type II collagen (CII; Chondrex) was emulsified with complete Freund's adjuvant (CFA) containing 4 mg/ml heat‐denatured mycobacterium (Chondrex, LLC) at a ratio of 1:1 (100 µg/mouse) and then injected intradermally into the tail (1.5 cm from the base) of DBA1/J mice to induce CIA (Day 0), followed by a booster immunization with 100 µg CII in incomplete adjuvant (IFA; Chondrex) at Day 21.^36^ At the moment of the boost (Day 21), GMSC (1 × 10^6^/mouse) and GMSC‐Exo (165 µg/mouse) were injected into CIA mice (*n* = 6) via the lateral tail vein. In the control group, mice received equal PBS injection (*n* = 6).

Mice were monitored twice weekly for signs of arthritis based on arthritis scores and paw swelling. Each paw was evaluated and scored individually using a 0 to 4 scoring system: 0 = no damage; 1 = paw with detectable swelling in a single digit; 2 = paw with swelling in more than one digit; 3 = paw with swelling of all digits and instep; and 4 = severe swelling of the paw and ankle.^15^ On Day 56, all mice were euthanized, and peripheral blood, spleen, axillary and mesenteric lymph nodes (LN), and limbs were collected for further studies.

### 2.6 Histology and immunohistochemical staining

The excised paw was fixed in 4% paraformaldehyde, decalcificated, gradually dehydrated, embedded in paraffin and sliced into 4‐μm‐thick sections. The sections were treated with haematoxylin and eosin (H&E) staining and immunohistochemical staining. For immunohistochemical staining, the tissue sections were depleted of endogenous peroxidase activity with methanolic H_2_O_2_, blocked with normal serum for 30 min, and incubated overnight at 4°C with primary antibody (NF‐κB P65, Cell signaling, #8242T and GAPDH, Absin Bioscience, abs100005), then incubated with HRP labelled secondary antibody (G1213, goat anti‐rabbit; Servicebio) for 50 min at room temperature, and then reacted by 3,3′‐diaminobenzidine (G1211, immunohistochemical kit DAB chromogenic agent; Servicebio). A negative isotype‐matched control was included in each run. The sections were counterstained with haematoxylin, and the slides were scanned by PerkinElmer Pannoramic MIDI Digital Slide Scanner (3D HISTECH Ltd). The nucleus of haematoxylin stained is blue, and the positive expression of DAB is brownish yellow. The area of brownish yellow in different groups was determined by counting four random fields per section using CaseViewer 6.0 software, and histochemistry scores (H‐Score) were calculated. H‐Score (H‐SCORE=∑(PI×I)=(percentageofcellsofweakintensity×1)+(percentageofcellsofmoderateintensity×2)+percentageofcellsofstrongintensity×3).^37^, ^38^

### 2.7 micro‐CT analysis

The excised paw was fixed in 4% paraformaldehyde. The high‐resolution micro‐computed tomography (micro‐CT) system (Skyscan 1276; Bruker Micro‐CT) was used to acquire imaging of the three‐dimensional bone. The scans were performed with entire single mouse foot having the following parameters: 20 μm voxel size at 55 kV, 200 μA, 386 ms integration time, 516 image slices. Image reconstruction was performed using graphics processing unit (GPU)‐based reconstruction software, GPU‐Nrecon. Ring artefact and beam‐hardening correction were performed with this software as well. Reconstructed cross‐sections were reoriented, and ROI was further selected. Volumes of metatarsophalangeal joint were used to quantify bone erosion as follows. Second to fourth metatarsal and phalangeal bones were segmented from others using a consistent image intensity threshold. Three volumes of interest were set with ±1 mm length in the distal and proximal direction from the centre of each metatarsophalangeal joint. These volumes of interest were oriented consistently based on the 3D longitudinal axis of the third metatarsal. The bone volumes of the three metatarsophalangeal joints were then calculated.

### 2.8 Flow cytometry analysis

For GMSC phenotype identification, antibodies directed against human CD34, CD44, CD45, CD73, CD90, CD105 and isotype IgGs were obtained from Biolegend. The single cell suspensions were stained according to the protocol provided by the manufacturer. For cell analysis, total splenocytes or lymphocytes collected from spleen or LN of CIA mice were incubated in PBS containing 0.2% bovine serum albumin (BSA) and 1 μl of fluorophore‐conjugated antibodies (0.5 mg/ml) specific for CD4, CD25 or respective isotype controls (Biolegend) at 4°C for 20 min. For intracellular staining, cells were stimulated with PMA (50 ng/ml), ionomycin (1 μg/ml) and brefeldin A (10 μg/ml) (Biolegend) for 4 h. Then, the cells were fixed and permeabilized with Perm/Fix solution (BD Bioscience). Finally, 1 μl of anti‐IFN‐γ/anti‐IL17A (Nos. 505807/506915; Biolegend) for Th1 and Th17 subsets, and Foxp3 (No.320007; Biolegend) for regulatory T cells (Tregs) was added for 30 min in the dark. After washing with buffer, cells were analysed using a BD FACSCelesta flow cytometer and analysed with FlowJo software (version 10.0).

### 2.9 ELISA assays

The peripheral blood from CIA mice was stood for 20 min and centrifuged at 1000 *g* for 10 min to obtain the serum. The levels of cytokines (IFN‐γ, IL‐17A, IL‐10, IL‐6 and TNF‐α) in culture supernatants or serum were quantified by enzyme‐linked immunosorbent assay (ELISA) kit (Biolegend) according to the manufacturer's instructions.

### 2.10 Western blot analysis

Total proteins were extracted from the paw of CIA mice, and their concentration was determined using a BCA assay (Beyotime). Protein samples were applied and separated on 10% NuPAGE gel (Invitrogen), followed by transferring onto PVDF membranes (Millipore Inc.). Membranes were blocked in 5% non‐fat dry milk and 0.1% Tween‐20 for 1 h, followed by incubation overnight with primary antibody against mouse NF‐κB p50 (Abcam; ab32360), NF‐κB p65 (Cell Signalling; #8242T), Act 1 (Santa Cruz Biotechnology; sc‐100647), TRAF6 (Immunoway; YT4720), IL‐17RA (Absin Bioscience; abs140681) and GAPDH (Absin Bioscience; abs100005) diluted at 1:1000 in blocking solution. Next, the HRP‐conjugated secondary antibody (Absin Bioscience) at a dilution of 1:8000 was used to incubate the membranes for 2 h. Immunoreactive proteins were visualized using Tanon high‐sig ECL Western blotting Substrate and Tanon 5200 multifunction laser‐scanning system (Tanon).

### 2.11 LC‐MS/MS analysis

Equal amounts of proteins from the paw of CIA mice were separated on SDS‐PAGE. The bands from SDS‐PAGE with temperature dependence were excised and incubated with a solution of 1% SDS and 1% b‐mercaptoethanol at room temperature for 1 h. The excised gel stripes were dehydrated, reduced, alkylated and digested with trypsin at 37°C for 16 h. Then, the peptides were delivered onto a nano RP column and eluted with gradient (50%–80%) acetonitrile (ACN) for 60 min at 400 nl/min. After that, the elute was injected into a Q‐Exactive mass spectrometry set in a positive ion mode and a data dependent manner with a full MS scan from 350 to 2000 m/z. High collision energy dissociation was employed as the MS/MS acquisition method.^39^, ^40^ Proteome Discoverer 1.4 (Thermo Fisher Scientific) was used to convert raw MS/MS data into a MGF format. The exported MGF files were searched with Mascot v2.3.01 against the uniprot 20180305 mouse database (17,021 sequences) with a typtic specificity, allowing two missed cleavage. Carbamidomethylation was considered as fixed modification whereas oxidation (M) and Gln‐>pyro‐Glu (N‐term Q) as variable modifications. The mass tolerances for MS and MS/MS were 15 ppm and 20 mmu respectively. Proteins with false discovery rates <0.01 were further analysed.

### 2.12 Statistical analysis

SPSS 16.0 was used to perform statistical analysis. Significance was assessed by one‐way analysis of variance (ANOVA) followed by LSD post hoc test. *p* Values less than 0.05 were considered as significant.

## 3 RESULTS

### 3.1 Isolation and characterization of human GMSC

Gingival mesenchymal stem cell showed a relatively higher proliferation rate and number of population doublings compared to bone marrow mesenchymal stem cells (BMSCs).^12^ In our study, GMSC were isolated from human gingival tissues and exhibited a characteristic fibroblastic morphology (Figure 1A). In osteogenic and adipogenic induction conditions, GMSC differentiate into osteoblasts and adipocytes as determined by Alizarin Red S and Oil Red O staining respectively. The results showed that calcium nodules inside osteoblasts were stained black and red (Figure 1B), and the hollow vesicular droplets inside adipocytes were stained red (Figure 1C), which verified the multipotent differentiation capacities of GMSC. We used flow cytometry to further verify the stem cell phenotypic markers of GMSC. The results depicted that GMSC were negative for CD34 and CD45, the surface marker of hematopoietic stem/progenitor cell and leukocyte, but positive for CD44, CD73, CD90 and CD105 (Figure 1D).

### 3.2 Isolation and characterization of GMSC‐Exo

**FIGURE 2:**
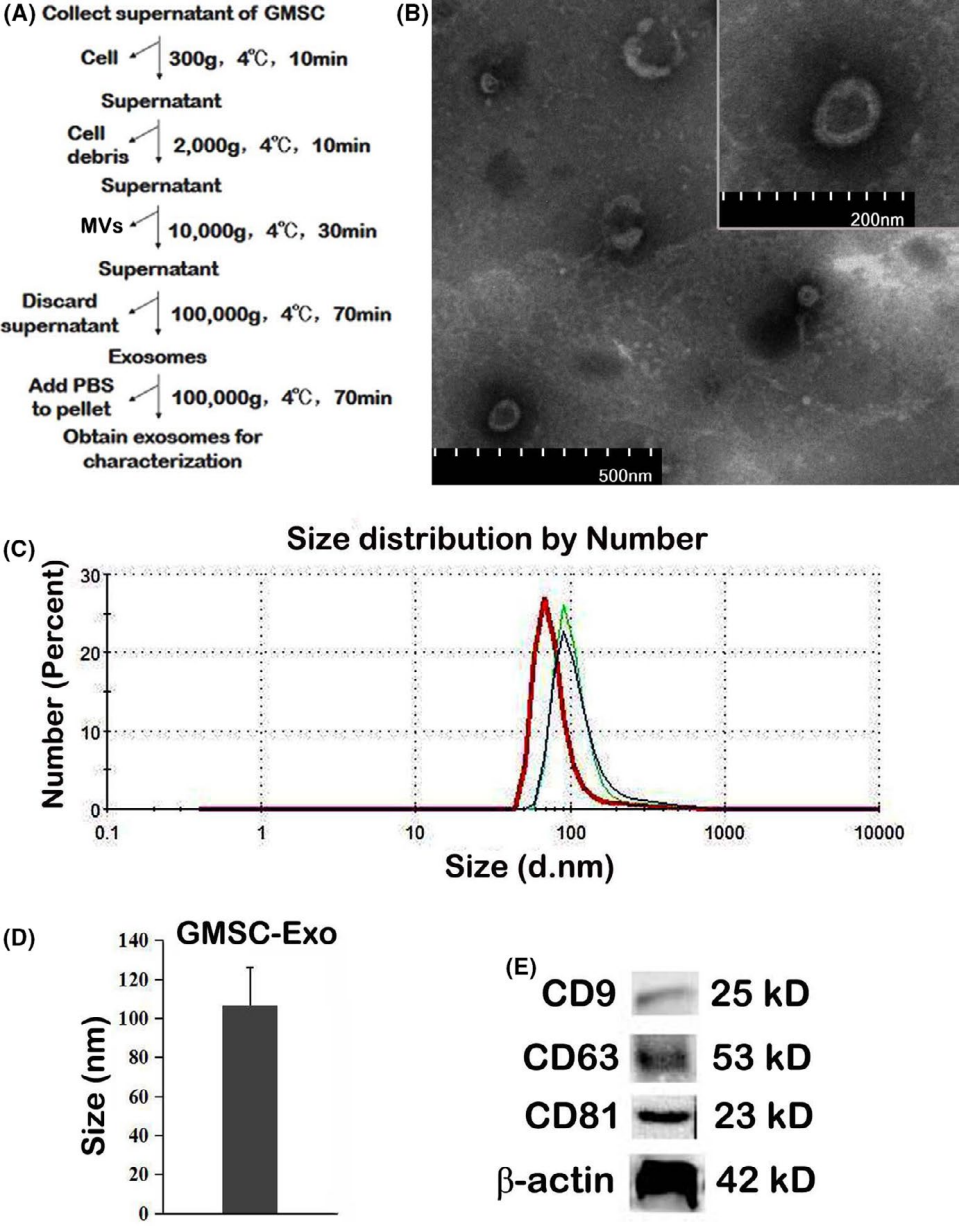
Isolation and characterization of GMSC‐Exo. (A) Experimental protocol for isolation of exosomes from GMSC‐conditioned medium using differential ultracentrifugation. (B) Representative images of GMSC‐Exo by TEM. Bars represent 500 nm in large picture and 200 nm for insert. (C) Number and size of GMSC‐Exo detected by nanoparticle tracking analysis. The analysis demonstrates that GMSC‐Exo have a single peak (~100 nm) diameter, and the mean diameter is 107 nm. (D) Mean size of GMSC‐Exo in the fractions represented in (C) (*n* = 3 biological replicates). (E) Detection of exosomal marker (CD9, CD63 and CD81) expression in purified GMSC‐Exo by Western blot. GMSC‐Exo, gingival mesenchymal stem cell‐derived exosomes; TEM, transmission electron microscopy

Gingival mesenchymal stem cell‐Exo were successfully isolated using the differential ultracentrifugation method as previously described^34^ (Figure 2A), and different tools were used to analyse them as recommended by the International Society for Extracellular Vesicles.^41^ TEM analysis showed that the round shaped vesicles were surrounded by a bilayer membrane (Figure 2B). The result of NTA analysis revealed the synthesis of three test data, all of which had single peaks (~100 nm) (Figure 2C). Our isolated GMSC‐Exo had the mean diameter of 106.88 ± 19.28 nm (Figure 2D). The results of Western blot analysis showed that GMSC‐Exo expressed the exosomal markers CD9, CD63 and CD81 (Figure 2E).

### 3.3 GMSC‐Exo exhibited stronger immunosuppressive effects than GMSC in vitro

**FIGURE 3:**
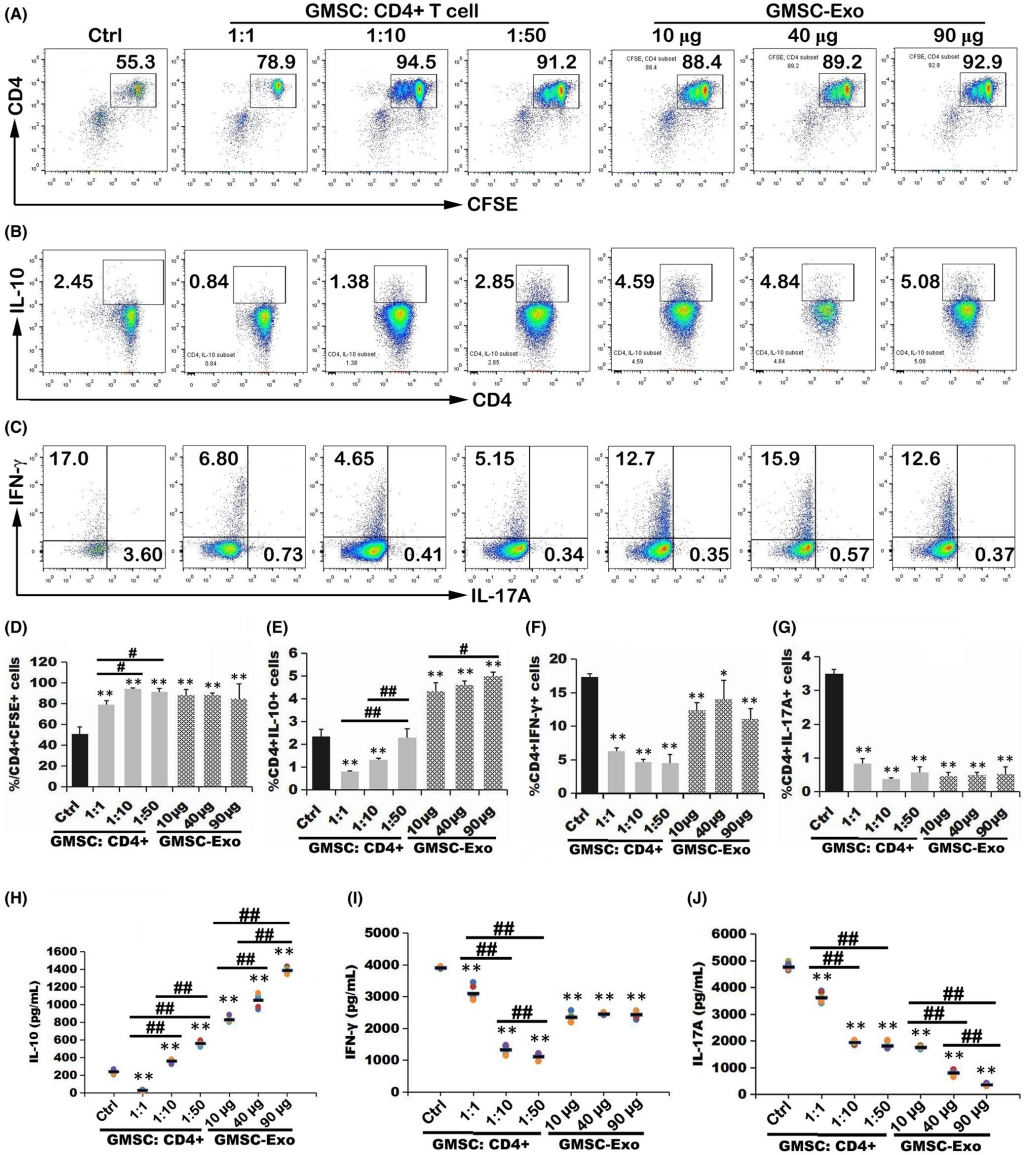
GMSC‐Exo exhibited immunosuppressive effects in vitro. CFSE‐labeled mouse CD4^+^ T cells were stimulated with soluble anti‐CD28 and co‐cultured with gradient doses of GMSC‐Exo or GMSC for 72 h. Then, the proliferation of CD4^+^ T cells or Th1, Th17 and Treg subsets was analyzed by flow cytometry (A‐G), and the cytokines (IFN‐γ, IL‐17A and IL‐10) were detected by ELISA methods from supernatant of co‐culture (H‐J). (A, B, C) The representative flow cytometry images show the expression of CFSE^+^CD4^+^, CFSE^+^CD4^+^IL‐10^+^Treg, CFSE^+^CD4^+^INF‐γ^+^Th1 and CFSE^+^CD4^+^IL‐17A^+^ Th17 cells. Data are representative of three separate experiments. (D‐G) Histograms show the percentages of CFSE^+^CD4^+^, CFSE^+^CD4^+^IL‐10^+^Treg, CFSE^+^CD4^+^INF‐γ^+^ Th1 and CFSE^+^CD4^+^IL‐17A^+^ Th17 cells after co‐culture with gradient doses of GMSC‐Exo or GMSC. Compared to control group (only CD4^+^ T cells, without co‐culture with GMSC or GMSC‐Exo): *p < 0.05, **p < 0.01. Comparison between groups treated with different doses of GMSC or GMSC‐Exo: ^#^ *p* < 0.05, ^##^ *p* < 0.01. After GMSC/GMSC‐Exo treatment, the proliferation of CD4^+^INF‐γ^+^ Th1 or CD4^+^IL‐17A^+^ Th17 cells was down‐regulated significantly, while that of CD4^+^IL‐10^+^Treg or CD4^+^ T cells was up‐regulated significantly. The data are presented as the mean ± SEM from three separate experiments. Data are representative of three separate experiments. (H‐J) The levels of IL‐10, IFN‐γ and IL‐17A were shown. After GMSC or GMSC‐Exo treatment, the level of IL‐10 was up‐regulated in a dose‐dependent manner, while that of IFN‐γ or IL‐17A was down‐regulated significantly. Compared to control group (only CD4^+^ T cells, without co‐culture with GMSC or GMSC‐Exo): *p < 0.05, **p < 0.01. Comparison between groups treated with different doses of GMSC or GMSC‐Exo: ^#^ *p* < 0.05, ^##^ *p* < 0.01. The data are presented as the mean ± SEM from three separate experiments. ELISA, enzyme‐linked immunosorbent assay; GMSC, gingival mesenchymal stem cells; GMSC‐Exo, gingival mesenchymal stem cell‐derived exosomes

We investigated the immunosuppressive properties of GMSC‐Exo and GMSC in a proliferation assay. Interestingly, the proliferation of CD4^+^ T cells increased after GMSC/GMSC‐Exo treatment (Figure 3A,D). However, GMSC‐Exo, but not GMSC, increased the percentages of CD4^+^IL‐10^+^ T cells in a dose‐dependent manner (Figure 3B,E). Both GMSC‐Exo and GMSC inhibited the percentages of CD4^+^IFN‐γ^+^Th1 and CD4^+^IL‐17A^+^Th17 subsets significantly (Figure 3C,F,G). We also measured cytokines in supernatant of co‐culture. After GMSC/GMSC‐Exo treatment, IL‐10 level was significantly up‐regulated and the change trend was consistent with that of CD4^+^IL‐10^+^ cells (Figure 3H). As expected, the levels of IFN‐γ and IL‐17A were significantly down‐regulated after GMSC/GMSC‐Exo treatment (Figure 3I‐J). Of interest, as the dose of GMSC‐Exo increased, the level of IL‐17A showed a dose‐dependent decrease, while IFN‐γ did not. These results indicated that in vitro, GMSC‐Exo exerted a stronger immunosuppressive effect than GMSC in vitro, which were likely due to decreased Th1/Th17 differentiation and generation of Treg cells.

### 3.4 GMSC‐Exo were efficient than GMSC in preventing mice from developing CIA

**FIGURE 4:**
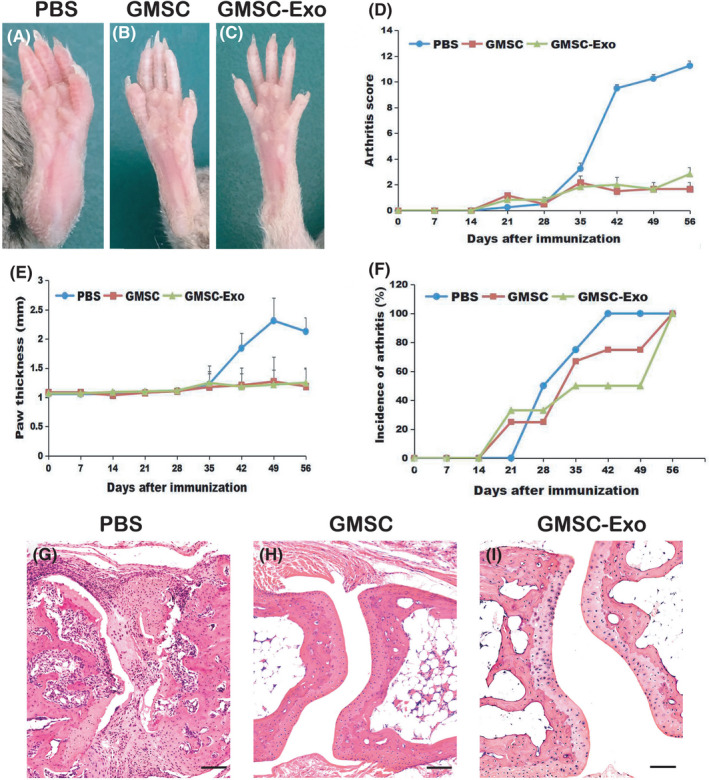
GMSC‐Exo alleviates the inflammatory responses in collagen‐induced arthritis (CIA) mice. CIA was induced in DBA/1 mice and after booster immunization at Day 21, mice were treated with GMSC‐Exo (165 µg per mouse, i.v.), GMSC (1 × 10^6^ per mouse, i.v.) or PBS, and euthanized on Day 56. (A, B, C) The representative images of gross appearance of swollen hind paws. (D‐E) Arthritis severity scores and paw thickness were determined at various time points after immunization. Compared with PBS‐treated group, arthritis scores in GMSC‐ or GMSC‐Exo‐treated group: **p < 0.01, ^##^ *p* < 0.01. Data are mean ± SD, *n* = 6 mice. (F) Incidences of CIA mice. *n* = 6 mice. (G, H, I) Histological analysis of whole ankle joints was shown. In PBS‐treated mice, inflammatory cells fill the joint cavity, and bone and cartilage are severely destroyed. After GMSC‐Exo or GMSC treatment, the degree of inflammation and bone destruction has been greatly improved. CIA, collagen‐induced arthritis; GMSC, gingival mesenchymal stem cells; GMSC‐Exo, gingival mesenchymal stem cell‐derived exosomes; PBS, phosphate‐buffered saline

To determine the immunomodulatory role of GMSC‐Exo in the context of autoimmune arthritis, we relied on the CIA model. We observed an evident reduction of joint swelling (Figure 4A‐C), markedly less severity of arthritis scores (Figure 4D), a significant reduction of paw thickness (Figure 4E) and decrease of incidence of arthritis (Figure 4F), following a single injection of GMSC‐Exo or GMSC on Day 21 after CII/CFA immunization. It should be noted that the incidence of arthritis is lower after GMSC‐exo treatment. Mice were sacrificed on Day 56 after CII/CFA immunization for histological analysis of whole ankle joints. The results indicated that the both GMSC‐Exo and GMSC could alleviate the severity of CIA, while GMSC‐Exo were more efficient than GMSC in preventing mice from developing CIA (Figure 4G‐I).

### 3.5 GMSC‐Exo have stronger effects than GMSC in preventing bone erosion in CIA mice

**FIGURE 5:**
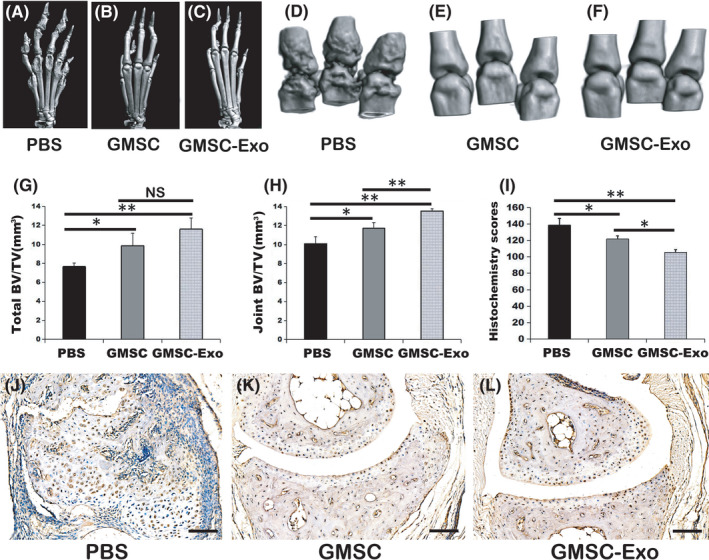
GMSC‐Exo suppresses bone erosion in CIA models. (A‐C) The representative 3D reconstruction images of ankle joints from different groups. (D‐F) The representative 3D reconstruction images of metatarsophalangeal joints from different groups. After GMSC or GMSC‐Exo treatment, the roughness of ankle joints or metatarsophalangeal reduced. (G) The total bone volumes of ankle joints. Data are mean ± SD, *n* = 6 mice. *p < 0.05, **p < 0.01. (H) The bone volumes of metatarsophalangeal joints. Both GMSC and GMSC‐Exo treatment reduced bone loss, and GMSC‐Exo gained better effect. Data are mean ± SD, *n* = 6 mice. *p < 0.05, **p < 0.01. (I‐L) The result of immunohistochemistry was shown. (I) The histochemistry scores were measured. After GMSC or GMSC‐Exo treatment, the scores reduced significantly, and GMSC‐Exo was better that GMSC. Data are mean ± SD, *n* = 6 mice. The animal experiments were performed in three independent experiments. *p < 0.05, **p < 0.01. (J‐L) The representative images of immunohistochemistry from different groups. The expression of NF‐κB p65/p105 in CIA synovium, bone and cartilage in PBS‐treated group was higher than in GMSC‐ or GMSC‐Exo‐treated groups. Bar = 100 μm. BV, bone volume; CIA, collagen‐induced arthritis; GMSC, gingival mesenchymal stem cells; GMSC‐Exo, gingival mesenchymal stem cell‐derived exosomes; PBS, phosphate‐buffered saline; TV, tissue volume

Bone erosion may develop as a result of cytokines released by inflammatory cells in the marrow. We used micro‐CT to evaluate the degree of bone erosion because it was better than histologic analysis for this aspect. The 3D images of PBS‐treated group showed that the surface of the cortical bone was rough, the bone structure of metatarsophalangeal and toe joints was irregular, and bone dissolution loss was significant (Figure 5A,D). In contrast, the surface of the cortical bone was smooth, the joint bone structure was neat and regular in GMSC (Figure 5B,E) or GMSC‐Exo‐treated group (Figure 5C,F). The volumes of whole paw or metatarsophalangeal joints from GMSC‐Exo‐treated mice were significantly more than those from PBS‐ or GMSC‐treated mice (Figure 5G‐H), which also indicated less bone degradation. The result of immunohistochemistry revealed that the expression level of NF‐κB p65/p105 in CIA synovium, bone and cartilage in GMSC‐Exo‐treated group was lower than in GMSC‐ or PBS‐treated groups (Figure 5I‐L). These data indicated that the GMSC‐Exo‐treated mice showed a lower degree of synovitis and destruction of bone as well as cartilage than GMSC‐ or PBS‐treated mice.

### 3.6 GMSC‐Exo and GMSC exhibited similar effects in increasing the percentages of Treg cells in vivo

**FIGURE 6:**
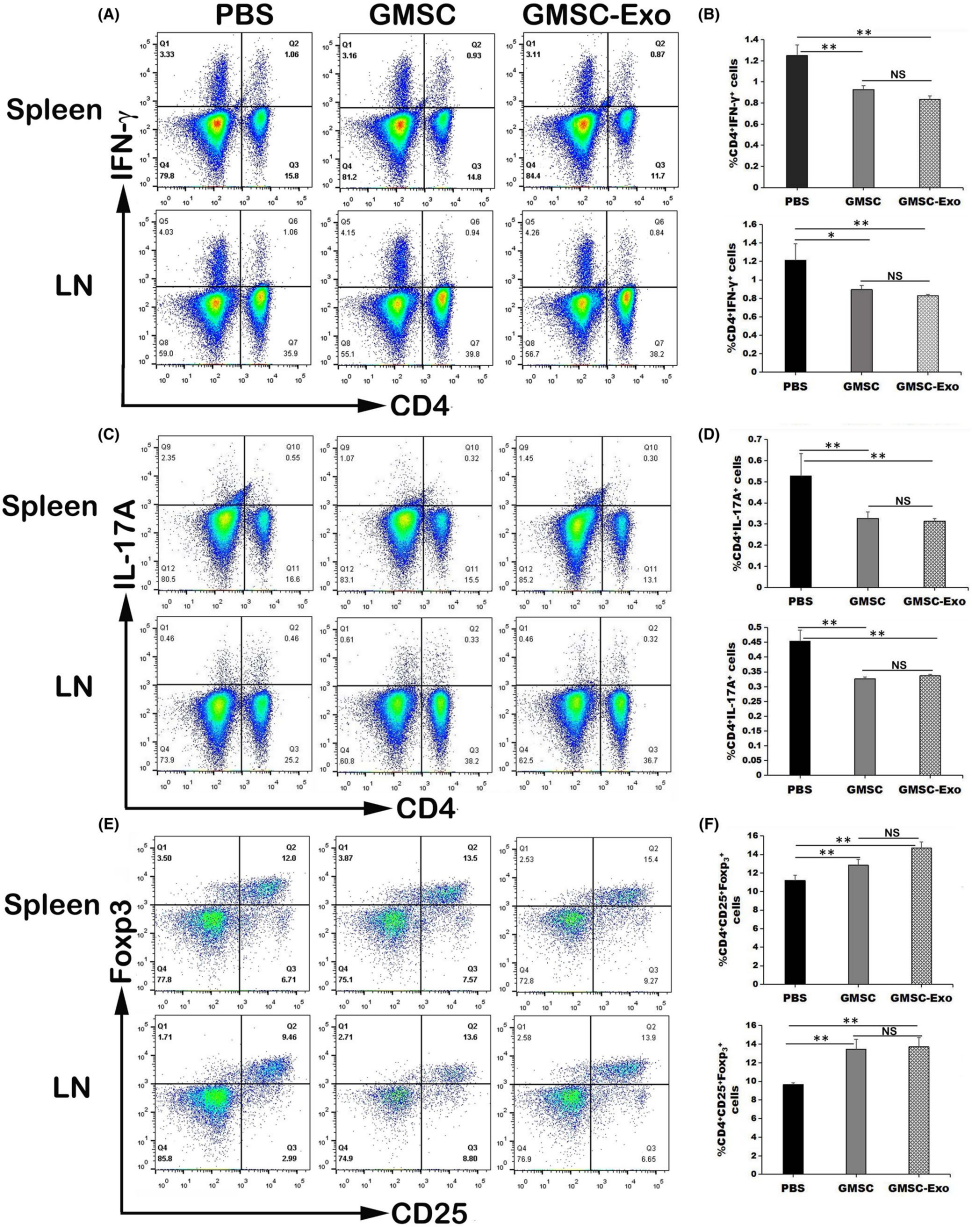
GMSC‐Exo therapy influences the polarization of Th cells in vivo. (A, C, E) Representative flow cytometry images show the expression of CD4^+^INF‐γ^+^ Th1, CD4^+^IL‐17A^+^ Th17 cells and CD4^+^CD25^+^FoxP3^+^ Treg in the spleens and LNs of three groups of CIA mice. (B, D, F) GMSC‐Exo and GMSC significantly decreased the percentage of CD4^+^INF‐γ^+^ Th1 and CD4^+^IL‐17A^+^ Th17 cells in the mouse spleen and LN, and increased the percentage of CD4^+^CD25^+^FoxP3^+^ Treg, whereas PBS did not significantly alter the frequencies of the subset Th cells in the CIA mice. *n* = 6 in each group. The animal experiments were performed in three independent experiments. *p < 0.05, **p < 0.01. Values are given as mean ± SD. CIA, collagen‐induced arthritis; GMSC, gingival mesenchymal stem cells; GMSC‐Exo, gingival mesenchymal stem cell‐derived exosomes; LNs, lymph nodes; NS, not significant; PBS, phosphate‐buffered saline

Due to the important role of Th1/Th17 cells and Treg cells in CIA progress, we next detected whether GMSC‐Exo treatment influence the percentages of CD4^+^ T‐cell subsets. The results of flow cytometric analysis showed that GMSC‐Exo or GMSC significantly decreased the percentages of CD4^+^INF‐γ^+^ Th1 (Figure 6A,B) and CD4^+^IL‐17A^+^ Th17 cells (Figure 6C,D), and increased the percentages of CD4^+^CD25^+^FoxP3^+^ Tregs (Figure 6E,F) in spleen and LN of the CIA mice, whereas the effect of GMSC‐Exo or GMSC had no significant difference. Thus, the GMSC‐Exo mediated immunoregulatory function in vivo may be attributed to increasing Tregs but also to other mechanisms.

### 3.7 GMSC‐Exo and GMSC exhibited similar effects on inhibiting the levels of pro‐inflammatory cytokines

**FIGURE 7:**
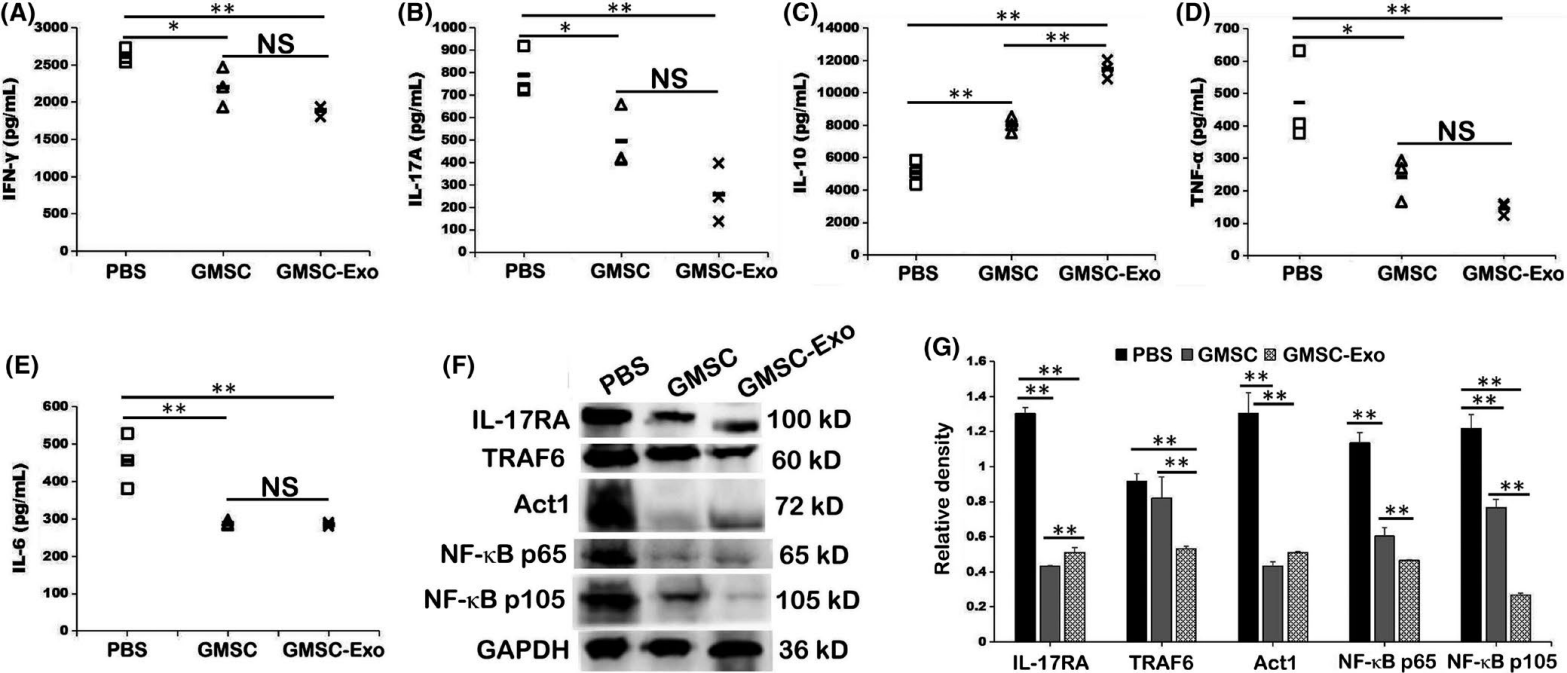
GMSC‐Exo reduces Th1 and Th17 cytokine production and increases Treg cytokine production in vivo, partially via IL‐17RA‐Act1‐TRAF6‐NF‐κB signal pathway. (A‐E) The expression of serum cytokines IFN‐γ, IL‐17A, IL‐10, TNF‐α and IL‐6 in CIA mice was determined by ELISA. Data are mean ± SD, *n* = 3 mice. (F) Total proteins were extracted from the paw of CIA mice after mouse sacrifice. The experiments were performed in three independent experiments. The main component proteins of IL‐17RA‐Act1‐TRAF6‐NF‐κB signal pathway expression were analyzed by Western blot. (G) The relative density of IL‐17RA, TRAF6, Act1 and NF‐κB p65/105 to GAPDH is shown by bar graph. One‐way ANOVA was used to compare different groups. Data are mean ± SD, *n* = 3 independent experiments. *p < 0.05, **p < 0.01. CIA, collagen‐induced arthritis; ELISA, enzyme‐linked immunosorbent assay; GMSC, gingival mesenchymal stem cells; GMSC‐Exo, gingival mesenchymal stem cell‐derived exosomes; NS, not significant; PBS, phosphate‐buffered saline

Meanwhile, we detected the systemic levels of pro‐inflammatory cytokines (IFN‐γ, IL‐17A, TNF‐α and IL‐6) and anti‐inflammatory cytokine (IL‐10) in serum of CIA mice. The results of ELISA showed that these pro‐inflammatory cytokines presented a drop tendency in mice that received GMSC‐Exo or GMSC treatment (Figure 7A,B,D,E), whereas the anti‐inflammatory cytokine presented an upward tendency (Figure 7C). These results illustrated that GMSC‐Exo mainly decreased the pathological inflammatory responses by reducing the levels of pro‐inflammatory cytokines and increasing that of anti‐inflammatory cytokine.

### 3.8 GMSC‐Exo alleviated CIA via IL‐17RA‐Act1‐TRAF6‐NF‐κB signal pathway

IL‐17 is a pro‐inflammatory cytokine mainly secreted by Th17 and other T cells, which plays a crucial role in many chronic inflammatory and autoimmune diseases. IL‐17 binds and acts through a heterodimeric receptor composed of two chains, IL‐17RA and IL‐17RC.^42^ Both chains contain a conserved signalling domain, the SEF/IL‐17R (SEFIR),^43^ which recruits the ubiquitin ligase Act1.^44^ Conversely, Act1 contains a TRAF‐binding domain, which recruits TRAF6,^45^ leading to the activation of NF‐κB, MAPK and PI3K pathway.^46^ Such activation results in excessive secretion of pro‐inflammatory cytokine and chemokine, including TNF, IL‐6, IL‐1β and IL‐8, leading to chronic inflammation and bone destruction. In our experiment, we found that GMSC‐Exo and GMSC could alleviate inflammation and prevent bone erosion in CIA mice. Therefore, we determined whether the immunosuppressive function worked through IL‐17RA‐Act1‐TRAF6‐NF‐κB signal pathway. The results of Western blot showed that both GMSC‐Exo and GMSC treatments inhibited the expression of IL‐17RA, Act1, TRAF6 and NF‐κB p65/p105 compared to PBS treatment (Figure 7F). In addition, GMSC‐Exo showed a better effect in suppressing TRAF6 and NF‐κB p65/p105. All differences are statistically significant (Figure 7G).

### 3.9 GMSC‐Exo‐treated group shared most of differentially expressed proteins and molecular function with GMSC‐treated group

**FIGURE 8:**
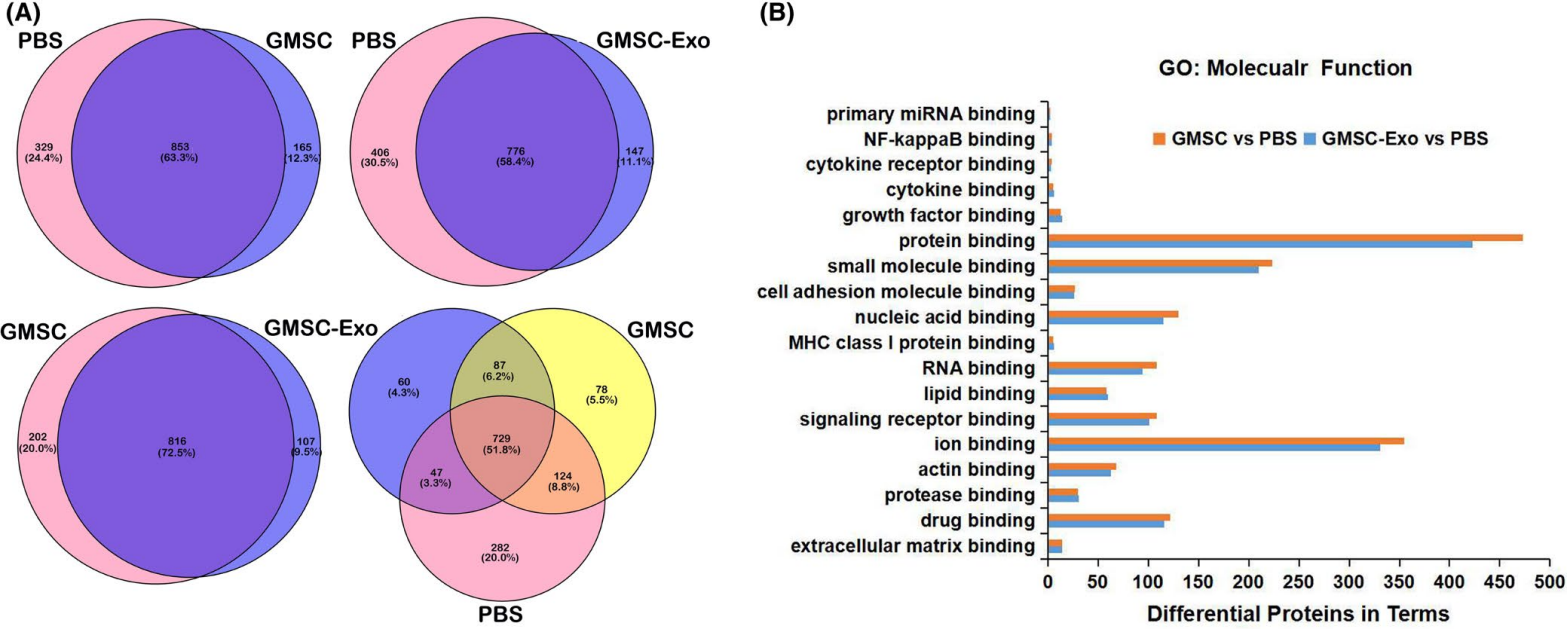
Bioinformatics analysis of the proteins identified in paws of CIA mice. (A) Venn diagram illustrates the overlap between the DEPs in paws of CIA mice treated with GMSC‐Exo, GMSC and PBS. As shown in the Venn diagram, a major fraction of DEPs was only shared between GMSC‐Exo and GMSC group. (B) Enrichment analysis of DEPs based on binding of GO annotation in terms of molecular function. GMSC‐Exo‐treated group share most of molecular functions of GMSC‐treated group in 18 kinds of binding functions. CIA, collagen‐induced arthritis; DEPs, differentially expressed proteins; GMSC, gingival mesenchymal stem cells; GMSC‐Exo, gingival mesenchymal stem cell‐derived exosomes; GO, gene ontology; PBS, phosphate‐buffered saline

To further clarify the immunosuppressive role of GMSC‐Exo in replacing GMSC, we performed global protein expression profiling of paws of CIA mice treated with GMSC‐Exo, GMSC and PBS. We identified 923, 1018 and 1182 differentially expressed proteins (DEPs) in GMSC‐Exo‐, GMSC‐ and PBS‐treated groups, respectively, and compared DEPs between two groups (Figure 8A). Out of 1018 DEPs identified in GMSC‐treated group, 816 DEPs (72.5%) significantly overlapped with the DEPs found in GMSC‐Exo‐treated group (Figure 8A), and the ratio was the highest among three comparisons, indicating that GMSC‐Exo‐treated group shared most DEPs with GMSC‐treated group. To reveal the biological function of the identified proteins, we performed gene ontology (GO) analysis based on molecular function using blast2go analytical software.^47^ We found 18 kinds of binding functions and on the top included protein binding, ion binding and small molecular binding. In addition, the binding of nucleic acid, drug and RNA were also ranked as top functions (Figure 8B). Interestingly, GMSC‐Exo‐treated group shared most of molecular functions of GMSC‐treated group, indicating the potential of GMSC‐Exo in replacing GMSC.

## 4 DISCUSSION

This study provides the first evidence that GMSC‐Exo exert immunomodulatory effect in CIA model. Moreover, this is one of the few studies reporting the role of GMSC‐Exo in vivo and in vitro.^24^, ^29^, ^30^, ^48^, ^49^

In order to verify whether GMSC‐Exo has the same immunosuppressive effects as GMSC and explore their therapeutical mechanisms, we carried out in vitro and in vivo experiments. In vitro, GMSC‐Exo/GMSC were co‐cultured with CD4^+^ T cells for 3 days, and the results show that the percentages of CD4^+^IL‐10^+^ T cells increased in a dose‐dependent manner (Figure 3B,E), while those of CD4^+^IFN‐γ^+^Th1 and CD4^+^IL‐17A^+^Th17 subsets decrease significantly (Figure 3C,F,G), which indicate that GMSC‐Exo/GMSC promote the polarization of peripheral Treg subsets but inhibit that of Th1 and Th17 subsets from CD4^+^ T cells. Interestingly, the percentages of CD4^+^ T cells increase (Figure 3A,D), which are consistent with the report of Sybren et al.^50^ This may be explained by mitochondrial transfer from MSCs to CD4^+^ T cells, which is a novel immunoregulatory mechanism.^51^ The mitochondrial transfer modifies the recipient cell bioenergetics and stimulates their proliferation and functions. In vivo, after systemic administration of GMSC‐Exo/GMSC, the inflammation and bone erosion of joints reduce significantly (Figures 4 and 5). In addition, the CD4^+^CD25^+^Foxp3^+^ Tregs also increase significantly, while CD4^+^IFN‐γ^+^Th1 and CD4^+^IL‐17A^+^Th17 subsets decrease markedly, both in spleen and LN (Figure 6). The above data indicate that GMSC‐Exo may exert immunosuppressive effects via regulating the interconversion of Th17/peripheral Treg subsets.

Undoubtedly, cytokines also play a key role in the pathogenesis of RA.^52^ They contribute to the induction or suppression of inflammation and thus provide therapeutic targets.^53^, ^54^ In this study, we found that the changes in the levels of cytokines are consistent with their corresponding immune cells both in vivo and in vitro. Results show that after GMSC‐Exo/GMSC treatment, the level of IL‐10 elevate while that of IFN‐γ and IL‐17A reduce significantly (Figures 3H‐J and 7A‐C). IL‐10 is an anti‐inflammatory cytokine produced by multiple cell types, including B cells, Th2 cells and Treg cells, inhibits neutrophil infiltration and activation as well as pathogenic Th17 cells in the synovial tissue, skews macrophage polarization towards an M2 phenotype and inhibits the expression of key pro‐inflammatory cytokines such as TNF‐α, IL‐1β and IL‐6.^54^ In addition, Th1 cells produce IFN‐γ, which has been reported to suppress inflammation through inhibition of Th17 responses.^55^ IL‐17 contributes to neutrophil recruitment, a hallmark of RA synovial fluid, and synovial IL‐17 from RA patients was shown to induce bone resorption.^56^ IL‐17RA and IL‐17RC are broadly expressed in whole synovium tissue from RA patients.^57^ They overexpress in synoviocytes and play an important role in RA pathogenesis, and also be found on CD14^+^ monocytes, CD3^+^ lymphocytes and CD19^+^ lymphocytes. This overexpression could make synoviocytes and immune cells more sensitive to IL‐17, a major regulatory cytokine implicated in RA pathogenesis.^57^ IL‐17A activates NF‐κB signal pathway by binding IL‐17RA, recruiting Act1, which contains a TRAF6‐motif, then activating p50 and p65, finally leading to the release of pro‐inflammatory factors (such as TNF‐α, IL‐1β). Our study indicates that GMSC‐Exo/GMSC infusion ameliorate inflammatory condition in CIA mice by suppressing pro‐inflammatory cytokines, promoting anti‐inflammatory cytokines, and then downregulating the activation of NF‐κB signalling (Figure 7F,G).

One of the key mechanisms of MSCs' anti‐inflammatory effects is the secretion of soluble factors with paracrine actions, which, at least in part, is mediated by exosomes.^26^, ^58^, ^59^ Exosomes are mainly released from the endosomal compartment and contain cargo including miRNA, mRNA and protein from the cell of their origin.^60^ In this study, we analysed the components of paws by LC/MS‐MS and found that GMSC‐Exo‐treated group shared most of DEPs and molecular function with GMSC‐treated group, which indicated that GMSC‐Exo has the potential to replace GMSC (Figure 8).

In this study, we compared the immunoregulatory effects of GMSC and GMSC‐Exo. The results showed that GMSC‐Exo have the same or even more immunoregulatory effects compared with GMSC in some aspects, such as promoting the secretion of IL‐10, inhibiting that of IL‐17A, and reducing the incidence of arthritis and bone erosion. To date, there are few reports that directly compare the effect of MSCs and MSC exosomes in the setting of CIA. We report for the first time that GMSC‐Exo have the same or better effects compared with GMSC in CIA. The reasons are speculated as follows: First, GMSC‐Exo share most of contents with GMSC, including microRNA, mRNA and proteins, which play a role in immunoregulatory process. Second, when injected intravenously, less than 1% of GMSC can reach the diseased site, differentiate into functional cells, or play a role through paracrine with the stimulation of the inflammatory microenvironment, while other GMSC especially which embedded in the capillary network of the lung, may have undergone apoptosis, although the apoptotic MSCs may participate in in vivo recipient‐mediated immunomodulation and exert a transient therapeutic effect.^22^, ^61^ Third, GMSC does not express MHC class Π molecules on the surface and cannot activate CD4^+^ T cells to trigger immune rejection, so it has low immunogenicity,^62^ but GMSC can express a small amount of MHC class I molecules, which allows it to be recognized and killed by CD8^+^ cells, thereby reducing its number. Therefore, GMSC‐Exo may have higher safety and more advantages in comparison with GMSC for preventing RA. However, the arthritis score is not significant at the time of treatment in our experiment, which means that mice have not developed arthritis when mice were treated. This is not really relevant in a clinical point of view, so the clinical application of GMSC‐Exo still needs further research.

## 5 CONCLUSION

This study compared the immunoregulatory effects of GMSC and GMSC‐Exo in CIA mice for the first time, and then explored the underlying mechanisms. The results showed that GMSC‐Exo have the same or better effects in comparison with GMSC in reducing the incidence of arthritis and bone erosion in CIA mice by regulating the imbalance of Th17/Treg and inhibiting IL‐17RA‐Act1‐TRAF6‐NF‐κB signalling pathway. Our studies have the potential to provide a promising cell‐free therapy for treating RA and other autoimmune diseases.

## CONFLICT OF INTEREST

The authors declare no competing interests.

## AUTHOR CONTRIBUTIONS

**Xiaohong Tian:** Conceptualization (lead); Data curation (lead); Formal analysis (lead); Methodology (lead); Project administration (lead); Writing – original draft (lead); Writing – review & editing (lead). **Wumei Wei:** Data curation (equal); Methodology (equal). **Yue Cao:** Data curation (equal); Methodology (equal). **Tianrang Ao:** Data curation (equal); Software (equal). **Feng Huang:** Project administration (equal); Resources (equal). **Rabia Javed:** Formal analysis (equal); Writing – review & editing (equal). **Xiaohong Wang:** Data curation (equal); Software (equal). **Jun Fan:** Data curation (equal). **Yanhui Zhang:** Methodology (equal); Software (equal). **Yanying Liu:** Data curation (equal). **Laijun Lai:** Data curation (equal); Writing – review & editing (equal). **Qiang Ao:** Conceptualization (equal); Funding acquisition (equal); Project administration (equal).

## Data Availability

The authors declare the data are available.
